# Finding a Composite Measure for Data From Wrist Actigraphy in Bipolar Disorder

**DOI:** 10.1093/geroni/igab046.1307

**Published:** 2021-12-17

**Authors:** Christopher Kaufmann, Ellen Lee, David Wing, Sonia Ancoli-Israel, Colin Depp, Ho-Kyoung Yoon, Lisa Eyler

**Affiliations:** 1 University of Florida, Gainesville, Florida, United States; 2 Department of Psychiatry, University of California San Diego, La Jolla, California, United States; 3 Center for Wireless and Population Health Systems, University of California San Diego, La Jolla, California, United States; 4 UC San Diego, La Jolla, California, United States; 5 Department of Psychiatry, Korea University College of Medicine, Seoul, Seoul-t'ukpyolsi, Republic of Korea

## Abstract

Actigraphy can objectively measure sleep in studies on Bipolar Disorder (BD) where subjective sleep ratings might be influenced by affect. Actigraphy data are complex necessitating data reduction approaches. We created a composite score of actigraphy sleep metrics (total sleep time [TST], wake after sleep onset [WASO], and percent sleep [PS]) in BD. We computed z-scores of sleep measures for n=51 BD vs. n=80 healthy subjects and averaged scores. We examined associations with participant characteristics and used LASSO to identify metrics best explaining composite variability. Higher composite scores (better sleep) were seen in employed vs. unemployed (t=2.40, df=34, p=0.02), and correlated with higher medication load (r=0.41, p=0.004), lower mania symptomatology (r=-0.33, p=0.04) and lower interleukin (IL)-6 levels (r=-0.32, p=0.02). TST best explained variability in medication load and PS best explained employment, mania symptoms and IL-6. Given observed specificity of associations, selecting theory-driven sleep metrics may be more appropriate than a composite.

